# A structural equation modeling study on factors influencing the humanistic care ability of undergraduate nursing interns

**DOI:** 10.3389/fmed.2026.1738203

**Published:** 2026-02-17

**Authors:** Jihong Wang, Yuxiao Wang, Meng Liu, Jinyan Li, Ju Qiu, Yufan Wang, Qihao Yang, Li Li, Xiaowei Wang, Fengxia Wang

**Affiliations:** Pingdingshan University, Pingdingshan, Henan, China

**Keywords:** communication ability, dual-mediation, emotional intelligence, humanistic care, nursing interns, organizational caring atmosphere, path analysis

## Abstract

**Objective:**

This study aimed to examine how organizational caring climate, communication ability, and emotional intelligence jointly relate to nursing interns’ humanistic care ability within a dual-mediation path-analytic framework.

**Methods:**

Using a convenience sampling method, 657 nursing interns from five tertiary first-class hospitals in China were selected as participants. Surveys were conducted using the Humanistic Care Ability Scale, the Organizational Care Atmosphere Scale, the Caring Ability Inventory Scale, and the Emotional Intelligence Scale.

**Results:**

All four variables were positively correlated (*p* < 0.01). The sequential indirect effect of Caring Atmosphere on Humanistic Care *via* Communication Ability and Emotional Intelligence was 0.105 (95% CI 0.07–0.15); the total indirect effect was 0.348 (95% CI 0.27–0.42).

**Conclusion:**

The humanistic care ability of nursing interns is influenced by the objective factor of care atmosphere and the subjective factors of communication competence and emotional intelligence.

## 1 Introduction

In 2022, the National Health Commission of China emphasized in the “National Nursing Career Development Plan (2021–2025)” that strengthening humanistic care in nursing is core to the development of the nursing profession and the health of the people, and must not be absent. The importance of humanistic care in nursing has long been recognized internationally. In 1997, the renowned American nursing theorist Jean Watson proposed her seminal Theory of Human Caring, positing that caring, manifested through loving human interactions, is the essence of nursing. Humanistic care ability refers to the competence of nurses to provide holistic care that respects patient dignity, demonstrates empathy, and addresses psychosocial needs alongside physical care (1). The ability to provide humanistic care is considered one of the most fundamental and crucial competencies in nursing practice (2). This ability depends on how accurately nurses can identify patient needs and implement appropriate nursing actions. Existing evidence suggests that enhancing nurses’ humanistic care ability can be associated with improved patient satisfaction, alleviation of psychological distress, and enhanced coping abilities (3–5).

The organizational caring atmosphere (6), defined in the healthcare context as the psychological experience of feeling cared for, concerned about, and looked after directly or indirectly within the work environment, has been proposed as a significant contextual factor influencing nursing behaviors. When nursing students are immersed in a caring atmosphere, it facilitates their perception of care, fosters a caring attitude, and enhances their caring ability (7, 8). Furthermore, effective communication is integral to nursing. Joyce Travel bee (9) in her Human-to-Human Relationship Model, stated that communication is a necessary condition for effective nursing, enabling nurses to understand patients, identify and meet their needs, and achieve nursing goals. The evolving healthcare model requires nurses not only to communicate effectively with patients but also to perceive their emotional changes, making Emotional Intelligence highly important in the cognitive, affective, and psychological development of nursing students (10).

Therefore, guided by relevant theories, this study proposes a conceptual model to be tested. The hypothesis is that the organizational caring atmosphere, as a primary external factor, may directly or indirectly influence nursing interns’ humanistic care ability. Concurrently, emotional intelligence, as a key internal factor, and communication ability, as a factor mediating interaction with patients and the external environment, are also posited to relate to humanistic care ability. To test this model, this study selected nursing interns to construct a path analysis model identifying factors influencing their humanistic care ability, aiming to provide a theoretical basis for optimizing clinical internships.

## 2 Participants and methods

### 2.1 Participants and sampling

A convenience sampling method was employed to select 657 undergraduate nursing students interning at five tertiary first-class hospitals in Henan Province, China between February 2025 and June 2025 as study participants. Inclusion criteria were: (1) full-time undergraduate nursing students; (2) having completed an internship duration of more than 1 month; and (3) providing informed consent and voluntarily participating in the survey. Ethical approval for the study was granted by the Institutional Review Board of Pingdingshan University (Approval No: 20250318), and informed consent was obtained from all participants.

A total of 687 questionnaires were distributed. After excluding 21 invalid questionnaires due to incomplete responses or obvious response patterns, 666 were retained for initial review. Upon further data checking, 9 additional questionnaires with missing key variable data were excluded, resulting in 657 valid questionnaires, yielding an effective response rate of 95.6%.

### 2.2 Methods

#### 2.2.1 Study instruments

① *General information questionnaire*: self-designed by the researchers, covering gender, age, whether the participant is the only child in the family, experience as a student leader (class monitor, club president), and participation in university club activities.
② *Caring ability inventory (CAI)*: developed by Nkongho (22), this 37-item scale comprises three dimensions: Knowing, Courage, and Patience. It uses a 7-point Likert scale ranging from 1 (“strongly disagree”) to 7 (“strongly agree”), with 13 items being reverse-scored. Total scores range from 37 to 259, with subscale scores ranging from 14–98 (Knowing), 13–91 (Courage), and 10–70 (Patience). Higher scores indicate stronger caring ability. In a pilot study involving 50 nursing students conducted prior to the formal survey, the Cronbach’s alpha for the total scale was 0.851, In the current study, Cronbach’s *α* was 0.851 for the total scale, and 0.78, 0.81, and 0.76 for the Knowing, Courage, and Patience subscales, respectively.
③ *Supportive communication scale (SCS)*: developed by Whetten et al. (11), this 20-item scale includes three dimensions: Coaching and Counseling, Providing Effective Negative Feedback, and Supportive Communication. It employs a 6-point Likert scale, with all items positively scored. Higher scores indicate better communication skills. In the current study, Cronbach’s *α* was 0.851 for the total scale, and 0.78, 0.81, and 0.76 for the Knowing, Courage, and Patience subscales, respectively.
④ *Organizational climate for caring questionnaire (OCCQ)*: developed by Hughes (12), this 30-item questionnaire consists of three dimensions: Exemplar, Dialogue, and Confirmation. It uses a 6-point Likert scale. In the current study, Cronbach’s α was 0.836 for the total scale, and 0.77, 0.75, and 0.72 for the Exemplar, Dialogue, and Confirmation subscales, respectively.
⑤ *Emotional intelligence scale (EIS)*: developed by Schutte et al. (13) based on the Salovey and Mayer emotional intelligence model, this 33-item scale comprises four dimensions: Perception of Emotion, Managing Own Emotions, Managing Others’ Emotions, and Utilization of Emotions. It uses a 5-point Likert scale, except for items 5, 28, and 33, which are reverse-scored. Total scores range from 33 to 165, with higher scores indicating higher emotional intelligence. In the current study, Cronbach’s *α* was 0.898 for the total scale, and 0.82, 0.80, 0.78, and 0.81 for the Perception of Emotion, Managing Own Emotions, Managing Others’ Emotions, and Utilization of Emotions subscales, respectively.

### 2.3 Data collection

Prior to the formal survey, a pilot study was conducted with 50 nursing interns to ensure the clarity and appropriateness of the questionnaire. For the main study, the purpose, content, and significance of the research were explained to potential participants through internship group chats. Anonymity and confidentiality were explicitly guaranteed. Following the acquisition of informed consent, an online questionnaire link was generated using “Wenjuanxing” (a widely used Chinese online survey platform, equivalent to Amazon Mechanical Turk or Qualtrics in function) and distributed to the internship groups of the five target hospitals. Participants required approximately 20 to 25 min to complete the questionnaire before submission.

### 2.4 Statistical analysis

To test the hypothesized conceptual model, path analysis—rather than full structural equation modeling (SEM)—was conducted in AMOS. Path analysis was chosen because all variables were observed scores and no latent constructs were estimated, thereby yielding a more parsimonious and adequately powered test of the proposed mediation chain. Standardized path coefficients (*β*) and their significance were examined, and the significance of indirect effects was assessed with bias-corrected bootstrap confidence intervals (5,000 samples). Model fit was evaluated with *χ*^2^/df, RMSEA, GFI, AGFI, NFI, RFI, IFI, and CFI against accepted thresholds. Two-tailed *p* < 0.05 indicated statistical significance.

### 2.5 Common method bias assessment

To assess the potential risk of common method bias due to the use of self-reported data, Harman’s single-factor test was performed. The unrotated factor analysis revealed that the first factor accounted for 28.7% of the total variance, which is below the critical threshold of 40%, suggesting that common method bias was not a severe threat in this study.

## 3 Results

### 3.1 Scores for humanistic care ability, organizational care atmosphere, communication ability, and emotional intelligence

As shown in Table 1, the total scores for the nursing interns were as follows: Humanistic Care Ability (122.589 ± 35.693), organizational care atmosphere (94.270 ± 18.352), Communication Ability (63.242 ± 18.352), and Emotional Intelligence (91.636 ± 22.743).

**Table 1:** Scores for humanistic care ability, organizational care atmosphere, communication ability, and emotional intelligence among nursing interns.

Project	Item	Score
Total human care score	37	122.589 ± 35.693
Cognition	14	46.958 ± 17.169
Courage	13	43.155 ± 16.032
Patience	10	33.695 ± 12.930
Organizational care atmosphere	30	94.270 ± 18.352
Exemplary	14	44.429 ± 14.986
Conversation	9	28.100 ± 9.351
Confirmation	7	22.130 ± 7.518
Communication skills total score	20	63.242 ± 18.352
Coaching and counseling	3	9.482 ± 3.425
Providing effective negative feedback	6	19.497 ± 6.606
Supportive communication	11	34.382 ± 12.129
Total emotional intelligence score	33	91.636 ± 22.743
Emotional perception	12	33.837 ± 10.587
Self-emotion management	6	22.634 ± 7.093
Understanding others’ emotions	7	16.340 ± 5.325
Emotion utilization	8	19.442 ± 6.415

### 3.2 Correlations among humanistic care ability, organizational care atmosphere, communication ability, and emotional intelligence

As presented in Table 2, the total scores for organizational care atmosphere, communication ability, emotional intelligence, and humanistic care ability were all positively correlated with each other (*p* < 0.01). Furthermore, all three dimensions of organizational care atmosphere, all three dimensions of communication ability, and all four dimensions of emotional intelligence were positively correlated with both the total score and the three dimensions of humanistic care ability (*p* < 0.01).

**Table 2:** Correlation coefficients among humanistic care ability, organizational care atmosphere, communication ability, and emotional intelligence (r).

Variable	Total human care score	Cognition	Courage	Patience
Organizational care atmosphere total score	0.443^**^	0.338^**^	0.332^**^	0.373^**^
Exemplary	0.345^**^	0.269^**^	0.249^**^	0.297^**^
Conversation	0.370^**^	0.276^**^	0.282^**^	0.295^**^
Confirmation	0.350^**^	0.255^**^	0.272^**^	0.310^**^
Communication skills total score	0.459^**^	0.337^**^	0.363^**^	0.387^**^
Coaching and counseling	0.374^**^	0.292^**^	0.277^**^	0.301^**^
Providing effective negative feedback	0.340^**^	0.239^**^	0.280^**^	0.284^**^
Supportive communication	0.404^**^	0.297^**^	0.318^**^	0.346^**^
Total emotional intelligence score	0.486^**^	0.384^**^	0.378^**^	0.374^**^
Emotional perception	0.388^**^	0.304^**^	0.301^**^	0.304^**^
Self-emotion management	0.364^**^	0.282^**^	0.284^**^	0.285^**^
Understanding others’ emotions	0.368^**^	0.295^**^	0.283^**^	0.290^**^
Emotion utilization	0.401^**^	0.310^**^	0.317^**^	0.297^**^

**p < 0.01.

### 3.3 Path analysis results

**Figure 1:**
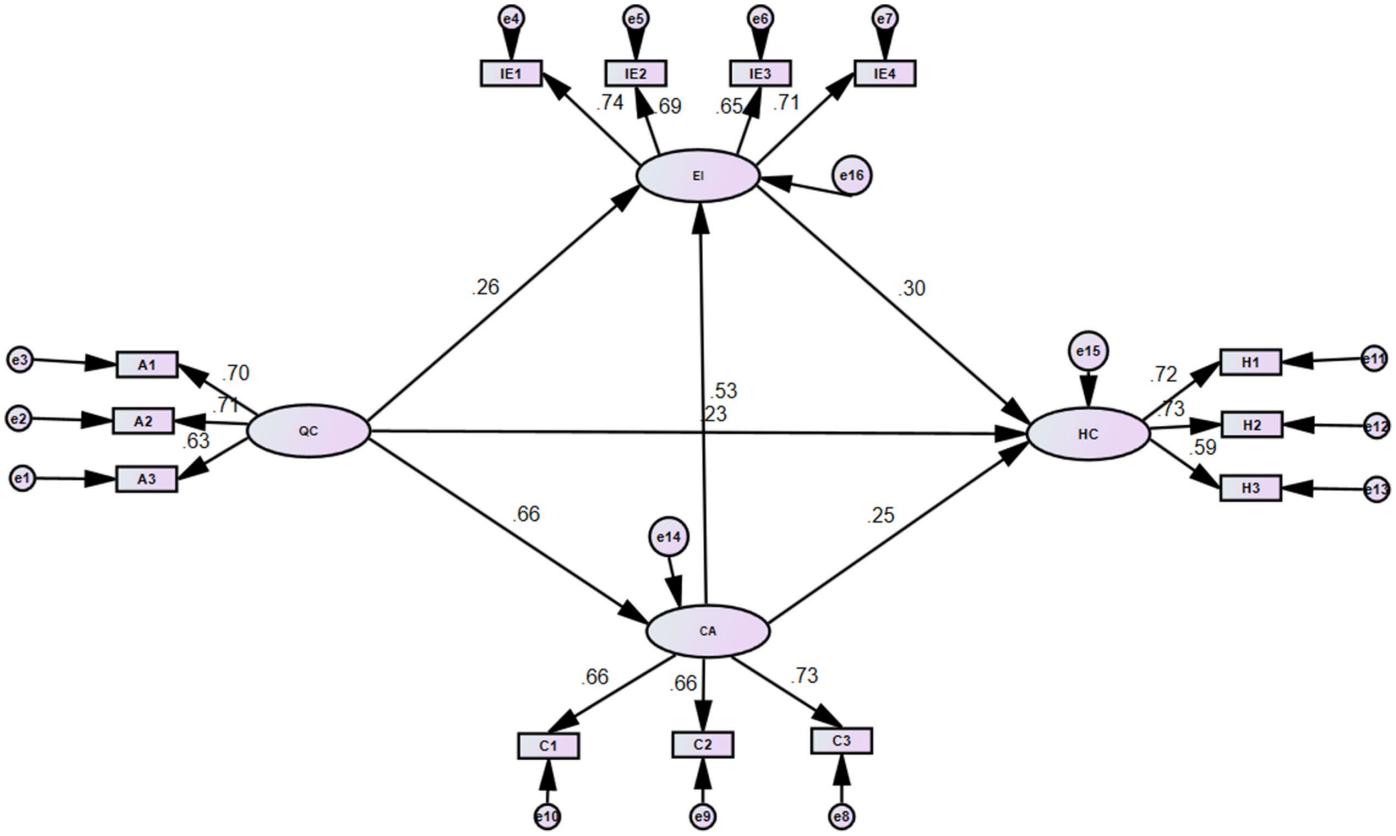
Path model of factors influencing humanistic care ability (with standardized coefficients). IE1, Emotional perception; IE2, Self-emotion management; IE3, Understanding others’ emotions; IE4, Emotion utilization; EI, Emotional intelligence; H1, Cognition; H2, Courage; H3, Patience; HC, Humanistic care; C1, Coaching and counseling; C2, Providing effective negative feedback; C3, Supportive communication; CA, Communication ability; A1, Exemplary; A2, Conversation; A3, Confirmation; QC, Caring atmosphere.

The hypothesized path model (Figure 1, Table 3) demonstrated acceptable fit to the data: *χ*^2^/df = 1.176, RMSEA = 0.016, GFI = 0.984, AGFI = 0.975, NFI = 0.974, RFI = 0.965, IFI = 0.996, CFI = 0.996, meeting recommended standards.

**Table 3:** Fitting metrics for the path model.

Indicators	*χ*^2^/df	RMSEA	GFI	AGFI	NFI	RFI	IFI	CFI
Fitting indicators	1.176	0.016	0.984	0.975	0.974	0.965	0.996	0.996
Reference standard	1–3	<0.05	>0.90	>0.90	>0.90	>0.90	>0.90	>0.90

As shown in Figure 1 and Table 4, organizational caring atmosphere had a significant direct effect on humanistic care ability (*β* = 0.23, *p* < 0.01). Communication ability and emotional intelligence also had significant direct effects on humanistic care ability (*β* = 0.25 and 0.30, respectively, *p* < 0.01). Furthermore, caring atmosphere significantly predicted both communication ability *(β* = 0.66, *p* < 0.01) and emotional intelligence (*β* = 0.26, *p* < 0.01), and communication ability significantly predicted emotional intelligence (*β* = 0.53, *p* < 0.01).

**Table 4:** Direct, indirect, and total effects in the path model (standardized).

Independent variable	Dependent variable	Direct effect (*β*)	Indirect effect (*β* [95% CI])	Total effect (*β*)
Organizational caring atmosphere	Humanistic care	0.23**	0.26 [0.18, 0.34]	0.49**
Organizational caring atmosphere	Emotional intelligence	0.26**	——	0.26**
Organizational caring atmosphere	Communication ability	0.66**	——	0.66**
Emotional intelligence	Humanistic care	0.30**	——	0.30**
Communication ability	Humanistic care	0.25**	——	0.25**
Communication ability	Emotional intelligence	0.53**	——	0.53**
Organizational caring atmosphere	→ Comm. ability → humanistic care		0.165 [0.11, 0.22]	
Organizational caring atmosphere	→ Emot. intelligence → humanistic care		0.078 [0.04, 0.12]	
Caring atmosphere	→ Comm. ability → emot. intel. → humanistic care		0.105 [0.07, 0.15]	

**p < 0.01; CI, Bias-corrected bootstrap confidence interval.

The indirect effect analysis revealed three significant mediation paths: (1) through communication ability alone (effect = 0.165, 95% CI [0.11, 0.22]), (2) through emotional intelligence alone (effect = 0.078, 95% CI [0.04, 0.12]), and (3) the sequential mediation through communication ability and then emotional intelligence (effect = 0.105, 95% CI [0.07, 0.15]). The total indirect effect was 0.348 (95% CI [0.27, 0.42]).

## 4 Discussion

### 4.1 Analysis of the current status of humanistic care ability, organizational caring atmosphere, communication ability, and emotional intelligence among nursing interns

The results of this study indicate that the humanistic care ability score of the nursing interns was 122.589 ± 35.693, which is consistent with findings from Wang et al. (14) and Machul et al. (15). Nursing education in China has long been influenced by a predominantly technical paradigm, leading to a relative lag in humanistic education. Although recent advocacy by nursing educators, both domestically and internationally, has prompted revisions of nursing curricula—including the addition of courses such as “Humanistic Cultivation for Nurses”—the humanistic care ability of the interns in this study remained moderate. This may be attributed to the fact that the interns, recruited from various universities across five teaching hospitals, had only entered clinical practice in June. Their relatively short internship duration, unfamiliarity with the hospital environment, and lack of proficiency in nursing procedures may have limited their capacity to provide holistic, caring nursing that addresses patients’ psychological and physiological needs.

The organizational caring atmosphere score among the interns was 94.270 ± 18.352, aligning with the results reported by Yoo et al. (16). As the participants were in the early stages of their internship, positive interactions among peers, patient mentoring from clinical instructors, and tolerance from patients and their families likely contributed to a strong perception of a caring atmosphere within the hospital.

The communication ability score was 63.242 ± 18.352, consistent with the findings of Bullington et al. (17). Most of the undergraduate interns surveyed had experience participating in club activities during their university studies. These activities provided opportunities to communicate with diverse individuals, thereby enhancing their communication skills and laying a foundation for more effective clinical practice.

The emotional intelligence score was 91.636 ± 22.743, which corresponds with results from Cichoń et al. (18). Nursing interns represent a unique group undergoing the transition from academic study to clinical practice. As all participants were full-time undergraduate students with years of higher education, they possessed mature psychological development and relatively good emotional self-regulation. Furthermore, over 93% of the sample was female. While this gender distribution reflects the regional nursing-student population, caution is needed in interpreting any gender-based associations. The observed emotional intelligence level should be understood within the specific context of this sample rather than attributed to gender stereotypes.

### 4.2 Correlation analysis among humanistic care ability, organizational caring atmosphere, communication ability, and emotional intelligence in nursing interns

The findings reveal a significant positive correlation between care atmosphere and humanistic care ability, indicating that a stronger care atmosphere is associated with enhanced humanistic care ability. Working in an environment characterized by care and support helps foster caring behaviors among interns. For those newly entering clinical practice, the care atmosphere is shaped by factors such as the supportive environment created by hospital administrators, the caring attitude of instructors toward patients, families, and interns themselves, and positive interactions among intern peers. However, the perception of this atmosphere is also determined by the individual’s own capacity for perception (19). Therefore, both academic educators and hospital managers should focus on enhancing the humanistic care atmosphere within clinical settings while simultaneously developing nursing students’ ability to perceive and internalize this care, thereby laying the groundwork for delivering compassionate nursing.

A significant positive correlation was also found between communication ability and humanistic care ability, suggesting that interns with stronger communication skills demonstrate greater humanistic care ability. The essence of humanistic care in nursing lies in compassionate human interaction, with effective communication being a vital component of delivering such care (20). During clinical practice, expressions of concern and caring for patients are largely conveyed through communication. Nursing schools should prioritize the development of communication skills by employing interactive teaching methods, such as scenario-based simulations and case studies, to increase student engagement and build a foundation for humanistic care ability. For students, actively participating in extracurricular clubs and engaging in communication with diverse groups can help translate theoretical knowledge into humanistic behaviors, thereby better preparing them for clinical communication.

Emotional intelligence also showed a significant positive correlation with humanistic care ability, implying that higher emotional intelligence is linked to greater humanistic care ability. Emotional intelligence is crucial for nurses working in emotionally charged environments, as it regulates their practice, interpersonal relationships, behaviors, and decision-making (21). Interns with higher emotional intelligence are better equipped to manage their own emotions in complex clinical situations, perceive the emotional states of patients and families, and implement targeted nursing interventions, ultimately enhancing patient satisfaction and fostering harmonious nurse–patient relationships.

### 4.3 The mediating pathways

The path analysis supports a model where the organizational caring atmosphere relates to humanistic care ability both directly and indirectly through communication ability and emotional intelligence. The sequential mediation path (Caring Atmosphere → Communication Ability → Emotional Intelligence → Humanistic Care) is theoretically plausible. In clinical settings, a supportive atmosphere may provide more opportunities for safe communication practice, which in turn could enhance interns’ awareness and management of emotions (their own and others’), ultimately contributing to humanistic care delivery. However, alternative model configurations are plausible and should be explored in future longitudinal research. The findings imply that interventions targeting the clinical environment, alongside training in communication and emotional skills, may be beneficial for fostering humanistic care.

### 4.4 Limitations and implications

This study has several limitations. First, the cross-sectional design precludes definitive causal inferences. The relationships discussed should be interpreted as associative. Second, the use of convenience sampling from one province in China limits the generalizability of the findings to other regions or cultural contexts. Third, despite statistical tests, the reliance on self-reported data may still be subject to biases. Fourth, the analysis used observed variable path analysis. Future research could employ latent variable SEM with multiple item parcels or indicators to better account for measurement error. Finally, alternative theoretical models were not tested; future studies should compare competing models.

Despite these limitations, the study offers theoretical and practical implications. Theoretically, it integrates organizational and individual factors within one model. Practically, it suggests that fostering a caring climate in teaching hospitals and incorporating targeted training on communication and emotional intelligence in nursing curricula could be valuable strategies. Future longitudinal or intervention studies are needed to verify these pathways and their outcomes on patient care.

## 5 Conclusion

This study indicates that the humanistic care ability of nursing interns is associated with the organizational caring atmosphere, communication ability, and emotional intelligence. Communication ability and emotional intelligence play mediating roles in this relationship. Therefore, a multifaceted approach addressing both the clinical learning environment and the development of interns’ interpersonal and intrapersonal competencies is recommended to support the cultivation of humanistic care ability.

## Data Availability

The original contributions presented in the study are included in the article/supplementary material, further inquiries can be directed to the corresponding author.
